# The campus healthy lifestyles, outcomes and experiences study: protocol for an observational assessment of college students’ health

**DOI:** 10.3389/fpubh.2025.1593943

**Published:** 2025-06-03

**Authors:** Alicia Anne Dahl, Stacy M. Fandetti, Trudy Moore-Harrison, Rosalba Barojas Chavarria, Maha Raya, Elizabeth F. Racine

**Affiliations:** ^1^Department of Epidemiology and Community Health, University of North Carolina at Charlotte, Charlotte, NC, United States; ^2^Department of Applied Physiology, Health, and Clinical Sciences, University of North Carolina at Charlotte, Charlotte, NC, United States; ^3^Texas A&M AgriLife Research and Extension Center at El Paso, El Paso, TX, United States

**Keywords:** college students, health behavior, risk assessment, chronic conditions, public health

## Abstract

As emerging adults transition from home to university campus living, they develop distinct health behaviors, including those surrounding food choice, exercise, and stress management. Concurrently, many college students experience body composition changes and weight gain at rates higher than those observed in the general population. Given that weight gain in college typically tracks into later life, a better understanding of the behavioral and physiological changes that occur during the first year of college is imperative. This protocol describes an observational study designed to assess behavioral and physiological changes among residential first-year college students with a university meal plan over one academic year (2023/2024). The protocol was developed through an iterative process incorporating research staff feedback and a pilot group sample from the previous academic year (2022/2023). Altogether, data were collected from 91 first-year college students at one university in the southeastern United States. Questionnaires were administered at three points in time, covering various individual health behavior topics (e.g., exercise, sleep, and substance use), nutrition literacy, diet quality, and campus food environment perceptions. Trained research assistants collected physical health measurements twice (approximately 6 months apart) at the university Health Risk Assessment Lab. These measurements included height, weight, blood pressure, a dual-energy x-ray absorptiometry (DEXA) scan, and a blood sample to evaluate markers of chronic disease risk (e.g., A1C and cholesterol). Participants also consented to share data on meal purchases and recreational center visits with their student identification cards. Several research questions will be explored with this data, including the association between health history and campus lifestyle behaviors and the physiological changes that occurred during the first year on campus.

## 1 Introduction

Emerging adulthood (18–24 years old) is a transitional life stage with increased independence and health decision-making. For many young adults, one significant life change is leaving home to attend college. In 2022, 39% of 18–24 year olds in the United States (US) were enrolled in college (1). Further, the percentage of emerging adults pursuing higher education has remained steady at nearly 40% for the past decade (1). Many changes occur during this life stage, including shifting interpersonal relationships and identity formation; therefore, establishing health-promoting habits is critical for chronic disease prevention and long-term health (2).

Existing research suggests that students typically gain weight during their first year of college, often called the ‘Freshman 15.’ A meta-analysis of longitudinal studies by Vadeboncoeur et al. (3) found that 60.9% of college students gained weight during their first year, with an average weight gain of 7.5 pounds. The rate of weight gain observed in the meta-analysis was five times higher than that observed at the population level at the time of the article’s publication (3). Interestingly, most of the weight gain occurred during the first semester (3). Another systematic review and meta-analysis found a modest but statistically significant increase of 3.5 pounds and a 1.2% increase in adiposity during a typical four-year college experience (4). The Fedewa et al. (4) study suggests unhealthy lifestyles persist through the entire college experience, not just during the first year, resulting in unfavorable weight gain and body composition changes.

Despite the known limitations of body mass index (BMI), the American College Health Association - National College Health Assessment (ACHA-NCHA) III cross-sectional survey results from spring 2024 show that 24% of students are categorized as being overweight (BMI 25–29.9 kg/m^2^) and 18% as obese (BMI ≥ 30 kg/m^2^), with the overall mean BMI of 25.60 and median of 24.13 (5). The prevalence of obesity among college students may be attributed to behavioral lifestyle factors such as changing dietary habits, physical activity levels, and stress associated with living independently in a new environment (6).

Changes in dietary habits, including food choice and consumption patterns (e.g., skipping meals, snacking), and physical activity are common among emerging adults, potentially leading to observed weight gain. According to the ACHA-NCHA, less than 30% of students reported eating three or more servings of vegetables, less than 20% consumed three or more servings of fruit, and most (70.6%) drank at least one sugar-sweetened beverage per day over 7 days (5). Poobalan et al. (7) found that most emerging adults did not meet dietary recommendations for fruits and vegetables, and approximately 60% consumed more than four unhealthy snacks daily. Another study of 269 first-year college students found that most exceeded the recommended U.S. dietary guideline limits for added sugar, refined grains, saturated fat, and sodium (8). Additionally, the literature indicates an association between unhealthy dietary patterns, food insecurity, and obesity (9). Among college students responding to the most recent ACHA-NCHA, 25.6% had low food security, and 21.0% had very low food security (5).

Emerging research suggests the COVID-19 pandemic added risk factors for unhealthy dietary behaviors. A 2021 systematic review found increased snack frequency, decreased fresh food, fruits, and vegetables intake, and an increased inclination for sweets and ultra-processed foods during and after the COVID-19 pandemic (10). Although this review was not specific to college students, the general trends toward unhealthier dietary habits during the pandemic are notable.

Aside from dietary patterns, other behavioral changes may increase the risk for chronic disease among college students. For example, students struggle to adhere to US national physical activity recommendations (11), with only 42.7% meeting aerobic and strength training guidelines in the spring 2024 semester (5). Also, given the academic and mental pressures associated with transitioning during the first-year experience, stress is a common sentiment among students. According to The American Institute of Stress, 45% of college students in the United States report experiencing “more than average stress,” and 23% of teenagers skip meals due to stress (12). This stress can impact college students’ sleep behaviors, with approximately 70% obtaining less than 8 h of sleep (13). Understanding more about the impact of the college environment on students’ health behaviors is warranted.

The aforementioned peer-reviewed literature on college students’ weight gain and food choices provides invaluable base knowledge, but most are nearly a decade old. Additionally, most studies assessing college students’ body composition or health behavior changes during the first year on campus are cross-sectional. Post-pandemic analyses are necessary to explore this emerging adult population and their health behaviors to understand them better and create opportunities for effective campus-wide interventions for chronic disease prevention. This is especially important for students who move away from home for the first time and are now free to make their own health behavior choices, which can potentially influence their long-term habits. Much of the extant literature is limited to cross-sectional and self-reported data. This paper describes the protocol of a longitudinal observational study to assess the physiological and behavioral health changes that occur during a college student’s first year of campus life through a combination of objective and subjective data.

## 2 Methods

### 2.1 Setting

This research study was conducted from 2022 to 2024 at a large public university (>30,000 students) in the Southeastern region of the United States (14). Undergraduate enrollment (>23,000) at this institution has been on a gradually increasing trajectory since the COVID-19 pandemic, serving 4,501 first-year students in 2023 (14). The first-year student population was diverse, with 39% identifying as minority; among those students, 17% identified as Black, 15% Hispanic, and 5% multiracial (14).

Health measurements were collected at the university’s Health Risk Assessment Lab (HRAL). The HRAL is a state-of-the-art space in the Department of [redacted for peer-reveiw] for researchers to assess biological health and screen for chronic disease. Measurement staff were required to complete several online courses including human subjects research training, First Aid & CPR training, Bloodborne Pathogens, a Laboratory Personnel Biosafety Basic Course, and radiation training. Next, staff received hands-on training from HRAL staff to operate the dual-energy x-ray absorptiometry (DEXA) scanner and collect biospecimens. The university Environmental Health and Safety department also required specific laboratory, research, and/or healthcare environment training. Once the required trainings were completed, the team developed a study safety protocol to protect staff and participants.

### 2.2 Design

The study design was intended to be observational without direct intervention during students’ first year living on campus. In 2022–2023, a pilot study was conducted to test and revise the recruitment protocol and measurements. In 2023–2024, we implemented a refined process. A total sample of 75 students was desired based on the measurement costs and available grant funds, recognizing less than 2% of the first-year student body would be included. The research questions to be explored include:

1. Is there an association between a history of chronic disease (e.g., diabetes, hypertension) and campus lifestyle behaviors?
2. What are the physiological changes [A1c, Cholesterol, blood pressure, BMI, body fat percentage, Visceral Adipose Tissue (abdominal fat)] of first-year college students in an academic year?
3. Is there an association between these health outcomes and campus lifestyle behaviors?
4. Is there a relationship between physical activity and meal-purchasing behaviors?
5. Is there an association between meal-purchasing behaviors, physical activity, and mental health?

The pilot study involved two measurement visits to the HRAL on campus: once in the fall semester and a second visit in late spring to capture baseline and end-of-year data. The same approach was used for the 2023/2024 study where students were asked to attend two in-person assessments at the HRAL: once in the fall semester and a second visit in late spring, with a goal of at least 6 months between visits. The pilot study approach was adapted to include a midpoint online behavioral survey in February 2024 to keep participants engaged and learn about temporal behavioral changes outside the beginning and end of the academic year.

Participants received up to $60 in Amazon.com gift cards for completing the measurement milestones: $25 for the HRAL visits and $10 for the online behavioral survey at the study midpoint. At the end of the study, students were emailed a summary report of their lab results across the academic year, with a recommendation to discuss the results with a healthcare provider. Also included in this email was a list of health-related campus and community resources to explore.

### 2.3 Recruitment

#### 2.3.1 Inclusion/exclusion criteria

To participate in the study, students were required to meet the following eligibility criteria: they must be a first-year undergraduate student, 18 years or older, not currently pregnant or planning to become pregnant within the next month, enrolled in a campus meal plan, and living on campus at the time of the study.

#### 2.3.2 Recruitment strategy

Various approaches were used to recruit eligible first-year university students, including in-person classroom presentations, flyers, email announcements sent to the university first-year listserv, faculty announcements in first-year courses, campus sidewalk chalk messages, and word of mouth by current research participants. Students interested in the research study completed a screening form to determine eligibility and provide contact information. Eligibility screening form items included age, current academic class standing, transfer status, student identification number, current meal plan participation, campus residency status, pregnancy status, recruitment method, and contact information. Ineligible students were notified of their status via email, and their data were permanently deleted from the screening database. For eligible students, a research team member reached out to schedule a phone call to discuss the study in detail and schedule the first measurement visit (T1) at the HRAL. Research staff contacted students up to three times via email and one time via text message to set up the initial phone call.

After the initial phone call to discuss study details, an electronic overview of the study and an informed consent form were emailed to the students to review before the first measurement visit. Each student was assigned a study identification number stored on a restricted Google drive. A reminder email with instructions was sent to each scheduled student the day before their visit, advising them how to prepare for the visit and what to bring.

### 2.4 Data collection

Data were collected at three points during the 2023–2024 academic year (see Table 1). The baseline time point (T1) was in the Fall 2023 semester (October and November) and consisted of physical health measurements and a behavioral survey. The second data collection point (T2) occurred in February 2024 and was an online survey only. The final time point (T3), which included physical health measurements and a behavioral survey, happened at the end of the Spring 2024 semester (April and May).

**Table 1:** Measurements across study time points.

Assessment measure/item	T1	T2	T3
Behavioral survey			
Demographics	X	X	X
Known medical conditions	X	X	X
Risk factors	X	X	X
USDA food security	X		
Health behaviors	X	X	X
Mental health	X	X	X
Brief cope	X	X	X
Diet quality questionnaire	X		X
Nutrition literacy		X	
University food environment			X
Physical health measurements			
Blood pressure	X		X
Height	X		X
Weight	X		X
DEXA (Total mass, body fat percentage, lean muscle mass, bone density, visceral fat, resting metabolic rate)	X		X
CardioCheck (HDL, LDL, total cholesterol, triglycerides, glucose)	X		X
A1C	X		X

Upon arriving at the HRAL, participants met with a trained measurement staff member who confirmed the student’s identity and ensured the consent form was completed. The student was then provided a study iPad to complete the health behavior questionnaire hosted on the university Qualtrics site, which took approximately 10–20 min to complete. Once the survey was completed, physical health measurements were collected.

#### 2.4.1 Survey measures

We selected survey measures that were validated among college students and reflected a range of behavioral factors related to the college experiences.

##### 2.4.1.1 American College Health Association (ACHA), National College Health Assessment (NCHA) III

The behavioral questionnaire included the following items borrowed from the ACHA questionnaire (NCHA III), which has been validated and broadly used across US colleges and universities: demographics (i.e., race, employment, first-generation college student status, gender, age), health history and pre-existing health conditions (i.e., chronic diseases, cancer, mental health), behavioral risk factors (e.g., tobacco use, alcohol consumption), self-reported weight-related behaviors and mental health. Reliability and validity data for the NCHA III is not yet available from the ACHA. Reliability and validity data for the NCHA III is not yet available from the ACHA.

##### 2.4.1.2 Brief Resilient Coping Scale (BRCS)

The Brief Resilient Coping Scale consists of 4 items on a five-point scale (“1” = does not describe me at all to “5” = describes me very well) measuring respondents’ coping tendencies (15). The total score can range from 4 to 20 (15). Questions included “I look for creative ways to alter difficult situations” and “I actively look for ways to replace the losses I encounter in life.” The BRCS has been shown to have adequate reliability and has been used with medical and nursing students (15).

##### 2.4.1.3 Young Adult Nutrition Literacy Tool (YA-NLT)

The Young Adult Nutrition Literacy is a validated tool developed to measure the three domains of nutrition literacy (functional, interactive, and critical) among college students (16). It consists of 42 items on a five-point Likert scale (“1” = strongly disagree to “5” = strongly agree) (16). The sum scores will be examined by domain and in total (total score range: 42–210). Questions include “I am aware of at least three of the 2020 Dietary Guidelines,” “I can plan healthy meals for a week,” and “I can identify credible sources of nutrition information.” The reliability of the YA-NLT was found to be similar to other nutrition literacy tools.

##### 2.4.1.4 USDA Household Food Security Survey Module (HFSSM)

The USDA Food Security survey reliably measures individual and household food insecurity, with the survey’s short form reducing participant burden while adequately identifying food-insecure individuals (17). The survey contains six items with three or four response options per question (e.g., “often true,” “sometimes true,” or “never true” for you in the last 30 days or “yes,” “no,” or “do not know”). We used a modified survey with four questions. Questions include “In the last 30 days, were you ever hungry but did not eat because there wasn’t enough money for food” and “the food that I bought just did not last, and I did not have money to get more.” For scoring purposes, responses of “often true,” “sometimes true,” and “yes” will be coded as “yes.” A raw score is then derived as a sum of the “yes” responses, with a raw score of 0–1 indicating food security and a score of 2–4 suggesting food insecurity. The survey has not been validated in the college student population, although it is frequently used due to its abbreviated form (18).

##### 2.4.1.5 Diet Quality Questionnaire (DQQ)

The Diet Quality Questionnaire contains 33 questions that can be answered with a “yes” or “no” regarding the foods consumed “yesterday” (19). Food categories include “fruit juice, fruit-flavored drinks, lemonade, or sweet tea” and “bread, rice, pasta, tortilla, or cereal.” The DQQ compares individual food group consumption patterns to global dietary recommendations to determine population-level adherence (20). The DQQ was determined to be valid at the population level in two large countries, although it has not yet been validated in the college student population (20). The DQQ provides several indicators of diet quality: the proportion of the population that consumed the five recommended food groups (ALL-5), non-communicable disease risk (NCD-risk) score, non-communicable disease protect (NCD-protect) score, and Global Dietary Recommendations (GDR) score. Scores are determined by adding the “yes” responses.

##### 2.4.1.6 University food environment questions

The survey also included eight questions about the university’s food environment: seven structured closed-response questions and one text entry question about money spent on food. These questions aimed to understand the students’ on-campus food purchasing habits and perceptions of the university food environment.

#### 2.4.2 Physical health measures

All anthropometric measurements were collected in the HRAL located on the university’s campus and physical assessment results were provided to participants at the end of the study, along with a campus resource list for consideration (e.g., campus dietitian, UREC group exercise weblink). Height and weight were measured via a medical-grade mechanical beam scale. Blood pressure was measured with a digital arm cuff. The blood collection for the cholesterol panel and A1C was done using a finger stick. The DEXA scan utilized two low-dose X-rays of two different energy levels (ionizing radiation) to measure lean tissue mass (muscle), bone mineral density, and total and regional body fat.

For the blood collection, the measurement staff technician wiped the participant’s finger clean with an alcohol pad and then pricked it with a lancet. The first drop was wiped with a gauze pad. A blood sample was loaded into the A1CNow + Professional Multi-Test HbA1c System to measure hemoglobin A1C. Then, additional blood was obtained via a capillary tube and test strip and processed using the PTS Diagnostics PTS760 CardioChek Plus Analyzer to measure total cholesterol, HDL, LDL, triglycerides, and glucose.

The DEXA scan was used for bone density and body composition analysis (GE Lunar Prodigy DEXA PR + 500,002-GA). The assessment included the following data points: body fat percentage, lean muscle mass, bone density, visceral fat, and resting metabolic rate. Bone density scans are often used to diagnose or assess the risk of osteoporosis, a health condition that weakens bones and makes them more likely to break, and early detection using DEXA is critical in identifying those at risk. A whole-body scan determined the body composition in the arms, legs, and trunk. Participants wore clothing without any metal in the fabric, and all jewelry was removed. Participants were instructed to lie face up on the scan table with arms at their sides and remain still. The DEXA arm scanned the body from head to toe, which took approximately 6–12 min. The scan utilizes two low-dose X-rays of two different energy levels (ionizing radiation) to measure lean tissue mass (muscle), bone mineral density, and total and regional body fat. Many health professionals consider DEXA the “gold standard” modality for assessing bone mineral density and body composition.

Research staff entered all data points into an online data collection form that was stored on a restricted study team drive. No identifying information was entered into the data collection form. For quality assurance purposes, a sample of the DEXA reports were cross-referenced with the measurements entered into the database to ensure consistency and accuracy in reporting.

#### 2.4.3 Data linkage

The survey responses and physical health measurements were linked to university-collected data regarding food purchases, university recreational center usage, and sociodemographic data via each student’s unique university identification (ID) number, which was removed from the dataset after pairing. Participants provided separate consent for the study to extract these data points from relevant university offices. The additional information the university collects will allow us to study purchasing behavior and physical activity levels trends and stratify the results by sociodemographic variables.

#### 2.4.4 Data confidentiality

Each participant received an assigned unique study ID number so the data collected were not assigned identifiable characteristics. This ID was used for their behavioral surveys, DEXA file names, and logs of measurement visits. The measurement team and the Principal Investigator were able to access the database on password protected devices. The Principal Investigator and Graduate Research Assistant maintained the only document with participant names and study ID to protect participant confidentiality.

### 2.5 Analytic plan

The normal distribution of data will be assessed using Kolmogorov–Smirnov and Shapiro–Wilk tests. For research questions that observe changes over the academic year, multiple regression analyses will be conducted for continuous variables (e.g., change in weight, body fat percentage, behavioral indicators) and logistic regressions will be applied to exploring dichotomous variables from the health history and health behavior measures (e.g., diagnosis, smoker status). Descriptive statistics will be used for cross-sectional data points (e.g., nutrition literacy). Group differences (e.g., gender, first-generation student status) will be assessed with ANOVA tests, where applicable. Multiple imputation will be applied where necessary for missing data. Hypotheses will be tested assuming a 0.05 significance level.

### 2.6 Ethical considerations

All study procedures were reviewed and approved by the Institutional Review Board. The informed consent procedure included providing students with a copy of the study details for review, with the opportunity to ask questions. Once consent was provided from the student, a research assistant (SF) and the Principal Investigator (AD) signed as witnesses. Physical copies of the consent form were made available in the HRAL.

The study protocol was updated to include an emergency plan and adverse event reporting to HRAL and university personnel. To provide more comfort to participants during the blood draw by finger stick, study staff added a fan, snacks, water to the space. For participants who experienced light-headedness, an additional step was included in the protocol: staff were to phone a participant’s friend to walk the student back to their dorm if they declined medical attention.

## 3 Results

**Figure 1:**
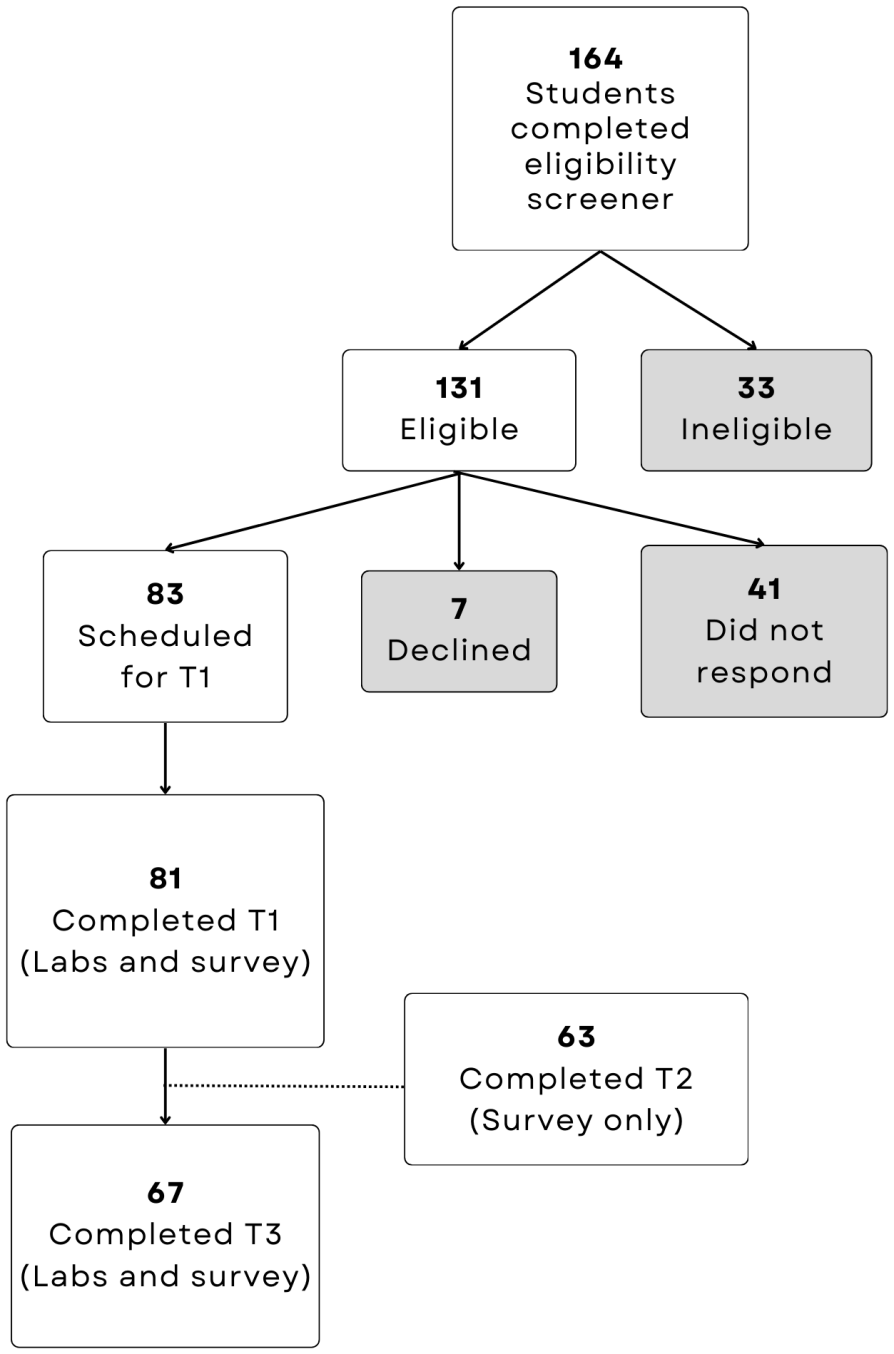
Flow chart of student recruitment and retention in the 2023/2024 academic year.

During the pilot study year (2022/2023), 24 students were recruited, 11 enrolled, 9 completed the baseline measurement visit, and five returned for the final visit. In the full study year, 164 students completed the eligibility screener, 83 enrolled, and 67 completed both HRAL visits (19.3% attrition). A participant flow chart is available in Figure 1.

### 3.1 Challenges

Notable issues arose during the implementation of our study. Institutional procedures delayed our timeframe for hiring and training measurement staff. We experienced several research administration delays related to budget approvals and ordering lab test kit supplies. Following the completion of data collection, we received a recall notice for the T3 A1C test, which may result in the inability to adequately report any observed changes to this outcome in the first year on campus. We were notified several months after data collection concluded, and the retesting of participants was not feasible due to funding and staffing availability at the end of the grant period.

The HRAL is a shared space, and appointments for different research studies overlapped occasionally, resulting in the need to reschedule participants. The DEXA machine was scheduled for maintenance during 1 week of our data collection period. The most common participant issue was an adverse reaction during the blood draw portion of the fall HRAL visit, resulting in fewer blood draws during the spring visit.

## 4 Discussion

Emerging adulthood is a critical period of growth and development. Within this age group, the literature indicates weight increases among two-thirds of first-year college students, and these physiological changes occur more rapidly than in the general population (3). The college experience offers unique opportunities around health and lifestyle behaviors. Without direct intervention, the current study aimed to monitor the physiological changes of first-year college students attending a public university in the Southeastern U.S.

Based on the implementation of this protocol, successful recruitment strategies that led to oversampling included university-wide study announcements distributed via email to all first-year students. Participant retention may have been improved by Amazon.com gift card incentives being distributed after each data collection point and the sharing of individualized study results to participants after completing the final study measurement visit. Establishing consistent data collection procedures over an academic year led to high retention rates for college students, which provided a foundation for testing various interventions and continued measurement over an extended period with this population. Future public health studies should consider longitudinal data collection to monitor college students’ physiological and behavioral changes and inform interventional approaches.

Residential universities are ideal for research trials or studying the effects of policy change (21). Students in a university environment typically remain in that environment for 4–5 years. This helps facilitate longitudinal studies, minimizing attrition (22). In addition, the majority of student services and systems are integrated at the student level (i.e., their student ID), making it relatively easy to link lifestyle behaviors (i.e., recreational use, dorm location, food purchasing behavior, health services utilization), with educational productivity (i.e., grade point average, course selection), and with demographic background information (i.e., financial status, work information, hometown information). This administrative data can be linked to primary data generated from the trial. This approach minimizes the respondents’ burden while maximizing the information the research team has to help interpret the results.

This study has several strengths, including the longitudinal exploration of objective physiological measures combined with self-reported and university-collected data. The dataset is robust. The work may be limited by students self-selecting into a health study, indicating a general interest in their health or well-being (23, 24). Since we did not stratify our sample, findings may not be generalizable to the broader student population. However, Ludy et al. (23) indicate that the self-selection of first-year students into health research studies may level out over time with comparators. Most importantly, our study sample comprised approximately 2% of the total enrollment of first-year students in the academic year. Now that we have evidence of effective recruitment strategies and a study protocol that led to high retention, we plan to scale this work to include an adequately powered sample of 350–450 participants. Future research may consider a longer-term follow-up beyond the first academic year on campus to explore whether time spent at a university increases health risks or serves as a protective factor for certain groups of students.

There are several implications of conducting an observational study on the physiological changes of first-year college students, paired with reports of health behaviors, across multiple time points. The findings may provide a clearer understanding of behavioral shifts over the academic year which can inform the delivery of health promotion strategies. When triangulated with physiological data collected in the lab, the exploration of university data about participants’ meal transactions and entrance swipes at recreation facilities on campus can provide evidence of behavioral patterns that increase or reduce risk for unfavorable health outcomes. Our findings may inform suggested revisions to meal plans or other institutional policies that are centered on student health. For example, if the first-year university meal plans are designed in a way that promotes a particular dining setting a number of times per day or week over others. Lastly, the forthcoming findings of this work may provide an updated understanding of student health behaviors and opportunities for designing tailored and interventions that respond to the health needs of first-year college students. This work has the potential to underscore the importance of comprehensive support systems for fostering healthy and successful transitions to college life.
